# Pathology of Hydrophobia

**Published:** 1876-04

**Authors:** 


THE PATHOLOGY OF HYDROPHOBIA.—In one of the late numbers of Virchow's *Archiv*, Dr. Benedikt states that he has examined a number of brains from rabid dogs, and the brain of a man who died from hydrophobia. The results which he obtained, and the conclusions he based upon them, are as follows: Hydrophobia is a poisoning of the blood, which being latent in the brain for awhile, breaks out at last. This takes place in certain parts of the brain. In dogs there were found changes in the olfactory gyrus of the anterior lobe, in a fossa which corresponds to the fissure of Sylvius in man, in the lenticular ganglion, and along the trifacial nerve toward its nucleus. The morbid changes seem to begin with a coagulation in the smaller veins, which is followed by increased pressure in the veins. In the course of the now-developing inflammation, the walls of the vessels are penetrated by red and white blood-corpuscles. The inner and middle coats of the vessels are torn in some places, while the outer coat probably becomes softened, and then a number of blood-corpuscles may leave the vessels by the openings thus formed. The blood-corpuscles form along the vessels a number of little abscesses in the brain substance. The corpuscles begin here to swell, and become at last transparent. The pre-existing cellular elements (of the brain) enclosed by the blood-corpuscles also swell, and become hyaloid. At last, blood-cells and brain-cells are softened, and changed into a transparent amorphous mass. The miliary abscesses in the brains of men are arranged in clusters around the vessels. There are also masses of yellow pigment along the vessels. These originate from red blood-corpuscles, which run through all stages of swelling. Only the nucleus is pigmented; there are also free nuclei. The appearance of the hallucinations and illusions, as well as of the motor form of psychosis in hydrophobia, is explained by the locality which becomes in this disease affected in the brain. The author does not deny that there may also be similar changes in a number of other organs.—*Medical and Surgical Reporter.*

